# Evaluation of the morphology and development of preantral ovarian follicles in mice submitted to a chronic diet of dietary supplementation with *Pereskia aculeata Miller* leaves

**DOI:** 10.1590/1984-3143-AR2024-0012

**Published:** 2024-07-15

**Authors:** Alesandro Silva Ferreira, Francisco Glauber Peixoto Ferreira, Etho Roberio Medeiros Nascimento, Gildas Mbemya Tetaping, Laritza Ferreira de Lima, Said Gonçalves da Cruz Fonseca, José Ricardo de Figueiredo, Daniel Freire de Sousa, Juliana Jales de Hollanda Celestino

**Affiliations:** 1 Instituto de Ciências da Saúde, Universidade da Integração Internacional da Lusofonia Afro-Brasileira – UNILAB, Redenção, CE, Brasil; 2 Programa de Pós-graduação em Desenvolvimento Rural, Universidade Federal do Rio Grande do Sul – UFRGS, Porto Alegre, RS, Brasil; 3 Laboratório de Manipulação de Oócitos e Folículos Ovarianos Pré-antrais – LAMOFOPA, Faculdade de Medicina Veterinária, Universidade Estadual do Ceará – UECE, Fortaleza, CE, Brasil; 4 Departamento de Farmácia, Universidade Federal do Ceará – UFC, Fortaleza, CE, Brasil

**Keywords:** animal feed, reproductive toxicity, ovarian follicles, medicinal plants, natural products

## Abstract

This study aimed to investigate the effect of including mouse feed with different concentrations (5, 10, or 20%) of *Pereskia aculeata Miller* (PAM) leaves on the morphology and development of preantral ovarian follicles and ovarian stromal cell density. The oral toxicity was performed using repeated dose toxicity assays subdivided into experiments of 30 days and 90 days of treatment. After the experiments, the ovaries of each animal were collected and submitted to classical histology. At 30 and 90 days, there was an equivalent percentage of normal, primordial, and developing follicles (P > 0.05) between PAM treatments compared to the control. Regarding the different stages of follicular development, after 90 days, there was a higher percentage (P < 0.05) of developing follicles only in the control group compared to day 30. The PAM 5% treatment was the only one that affected the cell density in the stroma after 90 days of treatment. Thus, we observed that supplementing the diet with *P. aculeata* did not pose any risk concerning animal consumption; specifically, there were no toxic reproductive effects observed from adding *Pereskia aculeata Miller* to the mouse diet.

## Introduction

Dietary manipulations promote effects especially justified by the use of energy as a variable since the energy density of the diet is one of the main factors involved in reproductive processes, such as ovarian follicular dynamics (Scaramuzzi et al., 2011). Among the plants used in human food, *Pereskia aculeata Miller* (*P. aculeata*) stands out, popularly known in English as Barbados gooseberry, and in Portuguese as ora-pro-nobis (Martin et al., 2017). This plant is considered an excellent food supplement because of its high values of protein, fiber, calcium, and iron, and is widely used and appreciated in various dishes of the regional cuisine, such as flour, salads, stews, pies, and pasta, such as noodles, as well as in home remedies (Almeida and Corrêa, 2012).

Although approached about the nutritional importance of *P. aculeata*, the plants still suffer a great restriction regarding the use and uptake, due to the reduced number of studies that showed its biological activity and safety regarding acute, chronic, or reproductive toxic effects (Sharapin, 1999). Among the safety criteria required are studies on reproductive toxicity. However, to date, there is no study investigating the effect of *P. aculeata Miller* leaves on the morphology and development of ovarian follicles.

Therefore, the present study investigated the effect of supplementing mouse feed with *P. aculeata* leaves on the morphology and development of preantral ovarian follicles and ovarian stroma cell density.

## Methods

### Site and collection of *P. aculeata*

The fresh leaves of *P. aculeta* previously identified in the Herbarium Prisco Bezerra of the Federal University of Ceará (UFC - n° 058870) were collected in the Vale da Biodiversidade located in the Massif of Baturité, Mulungu city in Ceara state (Longitude: 038°.31'.3928”, Latitude: 03°.44'.9775”, and Height: 14.587).

### Leaf and modified feed preparation

The fresh leaves of *P. aculeata* were previously dehydrated and crushed to obtain a sample with a farinaceous appearance. The *P. aculeata* meal (PAF) was added to the conventional feed previously ground and these components were mixed in an industrial blender until the complete homogenization, followed by the addition of carmellose (2%) as an aggregating agent. For this study, established concentrations were: 5%, 10%, and 20% of the inclusion level in the conventional feed (Fietz and Salgado, 1999; Almeida and Corrêa, 2012).

The material was pre-dried for 48 hours and subsequently, the material was extruded from the mass to make pellets, leaving it in the shape of conventional feed. The standard feed went through the same process, to simulate all the procedures done in the preparation of the modified feed.

### Animal and acclimatization

The animals used in the present study were female Swiss mice (n=60), 4 to 5 weeks old, weighing 27-32 g. These animals were placed in polypropylene boxes with wire lids, which were placed on shelves with controlled ventilation, maintained at a temperature of 22 ± 2 °C, with 12 h light/dark cycle, and received water and food *ad libitum*.

### Toxicity test at 30 and 90-day repeated doses

The oral toxicity using repeated doses following the Special Resolution (RE) No. 90 of the Brazilian National Health Surveillance Agency (ANVISA-Brazil) established repeated dose toxicity tests subdivided into four-week experiments (30 days of treatment) and twelve weeks (90 days of treatment).

The mice were distributed into four groups and organized into subplots of two groups, containing 7 and 8 animals for the 30 and 90-day tests, respectively. The four groups were composed of Control, that is, animals fed with Nuvilab CR-1 standard chow; the group with 5% (PAM5%), 10% (PAM10%), or 20% (PAM20%) of inclusion of *P. aculeata* flour.

### Preparation of histological slides and evaluation

After 30 and 90 days of treatment, the animals were sacrificed and their ovaries were subsequently fixed in 10% formalin for 48 hours, dehydrated in a graded series of ethanol, clarified in xylene, and embedded in paraffin for classical histology. The ovarian tissue was then sectioned serially at 7 µm intervals, and the sections were stained using the PAS-hematoxylin method. In the sections examined under an optical microscope, only the preantral follicles with visible oocyte nuclei were considered.

The developmental stages of follicles are primordial or growing follicles. These follicles were still classified individually as histologically normal when an intact oocyte was present, surrounded by granulosa cells that are well organized in one or more layers and that have no pyknotic nucleus. Atretic follicles were defined as those with a retracted oocyte, pyknotic nucleus, and/or disorganized granulosa cells detached from the basement membrane (Pedersen and Peters, 1968).

### Ovarian stromal cell density

For each treatment, 10% of the sections from each histological slide were randomly selected. In each tissue fragment, four distinct fields (2500 µm^2^) were chosen for cell counting using Image J 1.4.7 software (Alves et al., 2016).

### Statistical analysis

Data were processed by GraphPad Prism 5.0 software. The percentages of morphologically normal, primordial, and developing were initially subjected to the Smirnov-Kolmogorov and Bartlett tests to confirm normal distribution and homogeneity of variance, respectively. Data referring to ovarian stromal density were evaluated for normality using the Shapiro-Wilk test. Analysis of variance (ANOVA) was then performed by comparing means between treatments. Data were reported as mean (±SEM) and the results were considered significant when P<0.05.

### Ethical considerations in animal experimentation

This study was approved by the Ethics Committee in Animal Research (ECAR) of the Federal University of Ceara, Fortaleza-CE, under protocol and registration number No. 9/2016.

## Results

**Figure 1:**
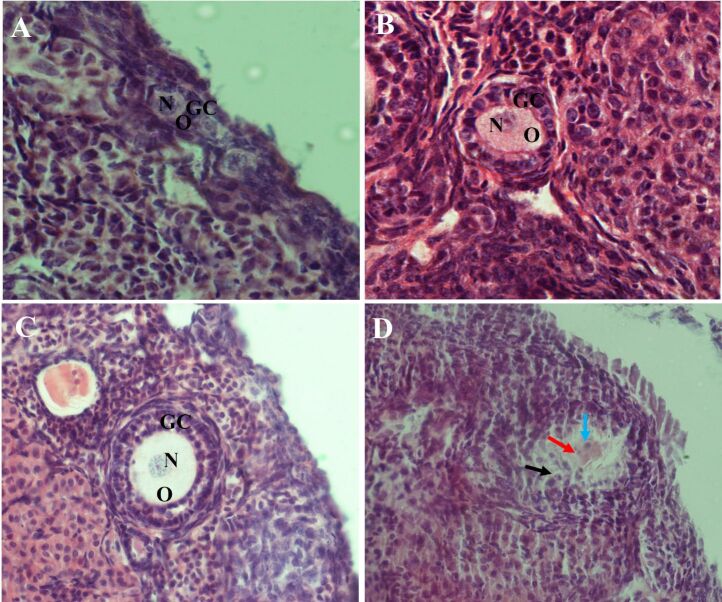
Representative images of morphologic aspects of ovarian follicles. Normal follicles are indicated in A (primordial), B (primary), and C (secondary). D represents degenerated follicle, the black arrow indicates disorganization of granulosa cells, the red arrow indicates oocyte retraction and the blue arrow indicates nuclear pyknosis. (O) oocyte; (N) nucleus of oocyte; (GC) granulosa cells. Scale bar = 50 μm.

A total of 1200 preantral follicles were evaluated in this study. Morphological aspects of ovarian follicles observed in the different treatments are shown in Figure 1.

**Figure 2:**
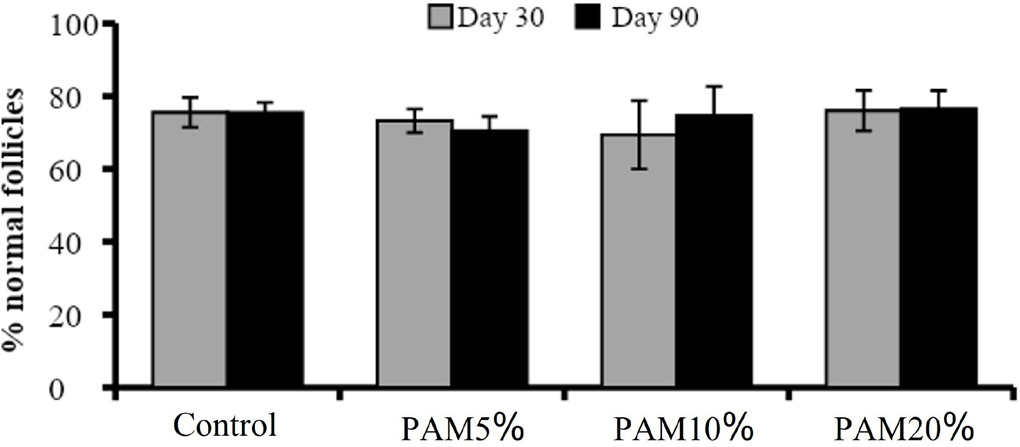
Percentage of normal follicles in the control and after consumption of *Pereskia aculeata Miller* flour (PAM) at different levels (5, 10, or 20%) during the period of 30 and 90 days.

Regarding the percentage of morphologically normal follicles in the control and the different concentrations of PAM flour inclusion, there was no statistical difference (P > 0.05) among the different treatments within the same period evaluated (day 30 or 90) or between the different periods (day 30 x day 90) within each treatment (Figure 2).

Table 1 shows the data regarding the percentage of primordial and developing follicles. In the established periods, there was no statistical difference (P > 0.05) among the different levels of PAM flour inclusion, and not even when compared to the control.

**Table 1:** Percentage of primordial and developing follicles after consumption of *Pereskia aculeata Miller* (PAM) flour during 30 and 90 days.

**Treatments**	**Primordial follicles**		**Developing follicles**	
	**Day 30**	**Day 90**	**Day 30**	**Day 90**
**Control**	47.97 ± 6.96 a	39.00 ± 3.94 b	52.03 ± 6.96 a	61.00 ± 3.94 b
**PAM5%**	48.05 ± 1.82 a	45.56 ± 2.68 a	51.95 ± 1.82 a	54.44 ± 2.68 a
**PAM10%**	46.00 ± 5,42 a	41.98 ± 5.45 a	54.00 ± 5.42 a	58.02 ± 5.45 a
**PAM20%**	47.79 ± 1.59 a	43.00 ± 13.11 a	52.21 ± 1.59 a	57.00 ± 13.12 a

^a,b^Different letters denote significant difference between days, within the same treatment and the same follicular category (P < 0.05).

When comparing the results between 30 and 90 days, within each treatment, we observed in the control after 90 days, a significant decrease (P < 0.05) in the percentage of primordial follicles, with a concomitant increase (P < 0.05) in the percentage of developing follicles. However, there was no difference (P > 0.05) in the different PAM inclusion concentrations (Table 1).

**Figure 3:**
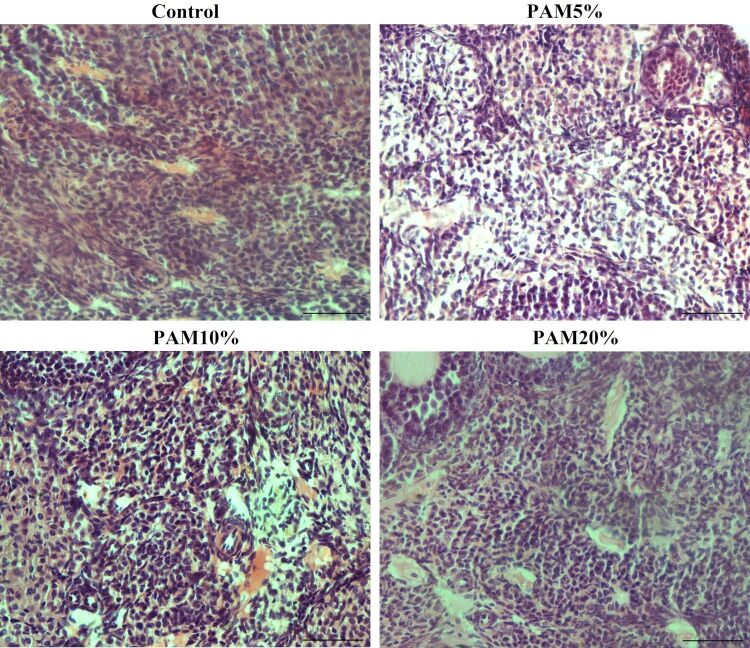
Representative images of stromal cell density of different treatments in the control and after consumption of *Pereskia aculeata Miller* flour (PAM) at different levels (5, 10, or 20%) during 90 days. Scale bar = 50 μm.

Figure 3 illustrates the cell density in the ovarian stroma of female mice observed in the control treatment and after 90 days of PAM flour consumption.

**Figure 4:**
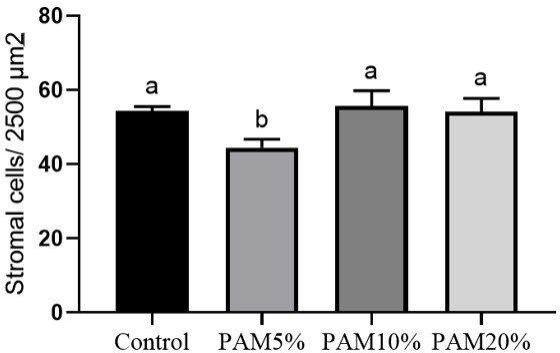
The mean number of stromal cells per 2500 µm^2^ in the control and after consumption of *Pereskia aculeata Miller* flour (PAM) at different levels (5, 10, or 20%) during 90 days. ^a,b^Different letters denote significant differences between treatments (P < 0.05).

As seen in Figure 4, PAM5% significantly reduced (P < 0.05) stromal cell density compared to the control and other concentrations of PAM after 90 days of treatment.

## Discussion

This study showed that the different inclusion concentrations of PAM administered to female Swiss mice did not affect the morphology of preantral follicles. According to Agostini-Costa (2020), the acute treatment of female Wistar rats with high doses of *P. aculeata* leaves extract showed no cutaneous, neurological, or behavioral changes in the animals, and especially, no changes in tissues or body and organ weight. The topical application of *P. aculeata* leaf extract in the acute dermal irritation test showed no clinical signs of local or even systemic, toxicity in the rats. *P. aculeata* leaves are commonly used as food in traditional Brazilian cuisine and as a topical remedy in folk medicine, and there are no reports of skin or systemic adverse effects (Pinto et al., 2015).

Regarding follicular development, there was a significantly higher percentage of developing follicles in the control group on day 90 compared to day 30, which was not observed in any of the PAM concentrations. Related to this, it is important to emphasize that phytoestrogens can potentially modulate cellular signaling pathways involved in follicular activation (Zhao and Mu, 2011). In this sense, studies have shown that metabolites such as isorhamnetin (Gao et al., 2017) and quercetin (Li et al., 2021), the main flavonoids present in *P. aculeata*, can inhibit the activation of the PI3K/Akt pathway, essential for follicular activation in mammals (Hsueh et al., 2015).

We observed that only the lowest concentration of *P. aculeata* flour (5%) affected stromal cell density after 90 days of treatment, reducing it in comparison to the other treatments. Mbemya et al. (2017) and Ñaupas et al. (2021) showed that the number of cells present in the ovarian stroma is positively related to the maintenance of morphological integrity and progression of follicular development. The reduction in cell density observed in PAM5% treatment did not affect morphological integrity. Just as it did not promote activation and follicular development, the inclusion of 5% PAM does not seem to have allowed for stromal cell proliferation, although there are no known mechanisms.

## Conclusion

The inclusion of *P. aculeata* leaves in the feed did not show any risk regarding animal consumption, since the analysis methods did not certify any type of toxic alteration on the morphology of the ovaries of the Swiss mice studied. Ensuring the food safety margin of the *P. aculeata Miller* plant, it becomes a nutritional alternative, contributing to the use of this species in both animal and human food. Besides this, the need to expand scientific research on this plant is ratified, including *in vitro* tests, assuring its important therapeutic use, including its anticancer potential.

## References

[B001] Agostini-Costa TS (2020). Bioactive compounds and health benefits of Pereskioideae and Cactoideae: a review. Food Chem.

[B002] Almeida MEF, Corrêa AD (2012). Utilização de cactáceas do gênero Pereskia na alimentação humana em um município de Minas Gerais. Ciência Rural.

[B003] Alves KA, Alves BG, Gastal GDA, Tarso SGS, Gastal MO, Figueiredo JR, Gambarini ML, Gastal EL (2016). The mare model to study the effects of ovarian dynamics on preantral follicle features. PLoS One.

[B004] Fietz VR, Salgado JM (1999). Efeito da pectina e da celulose nos níveis séricos de colesterol e triglicerídeos em ratos hiperlipidemicos. Food Sci Technol.

[B005] Gao L, Yao R, Liu Y, Wang Z, Huang Z, Du B, Zhang D, Wu L, Xiao L, Zhang Y (2017). Isorhamnetin protects against cardiac hypertrophy through blocking PI3K–AKT pathway. Mol Cell Biochem.

[B006] Hsueh AJ, Kawamura K, Cheng Y, Fauser BC (2015). Intraovarian control of early folliculogenesis. Endocr Rev.

[B007] Li J, Long H, Cong Y, Gao H, Lyu Q, Yu S, Kuang Y (2021). Quercetin prevents primordial follicle loss via suppression of PI3K/Akt/Foxo3a pathway activation in cyclophosphamide-treated mice. Reprod Biol Endocrinol.

[B008] Martin AA, Freitas RA, Sassaki GL, Evangelista PHL, Sierakowski MR (2017). Chemical structure and physical-chemical properties of mucilage from the leaves of Pereskia aculeata. Food Hydrocolloids.

[B009] Mbemya GT, Guerreiro DD, Donfack NJ, Silva LM, Vieira LA, Sousa FGC, Alves BG, Izaguirry FWS, Telefo FB, Pessoa ODL, Smitz J, Figueiredo JR, Rodrigues APR (2017). Justicia insularis improves the *in vitro* survival and development of ovine preantral follicles enclosed in ovarian tissue. J Pharm Pharmacol.

[B010] Ñaupas LVS, Brito DCC, Souza SS, Brandão FAS, Silva RF, Raposo RS, Moreira ACOM, Araújo AA, Alves BG, Guedes MIF, Silva JYG, Cordova A, Figueiredo JR, Rodrigues APR (2021). Alpha lipoic acid supplementation improves ovarian tissue vitrification outcome: an alternative to preserve the ovarian function of Morada Nova Ewe. Reprod Sci.

[B011] Pedersen T, Peters H (1968). Proposal for a classification of oocytes and follicles in the mouse ovary. J Reprod Fertil.

[B012] Pinto NDCC, Machado DC, Silva JM, Conegundes JLM, Gualberto ACM, Gameiro J, Cheidier LM, Castañon MCMN, Scio E (2015). *Pereskia aculeata Miller* leaves present in vivo topical anti-inflammatory activity in models of acute and chronic dermatitis. J Ethnopharmacol.

[B013] Scaramuzzi RJ, Baird DT, Campbell BK, Driancourt MA, Dupont J, Fortune JE, Gilchrist RB, Martin GB, Mcnatty KP, McNeilly AS, Monget P, Monniaux D, Viñoles C, Webb R (2011). Regulation of folliculogenesis and the determination of ovulation rate in ruminants. Reprod Fertil Dev.

[B014] Sharapin N (1999). Medicinal plants: pharmacopoeia prescriptions. An Acad Bras Cienc.

[B015] Zhao E, Mu Q (2011). Phytoestrogen biological actions on Mammalian reproductive system and cancer growth. Sci Pharm.

